# Supplementary material for: Neuromechanisms and subjective experiences during human-dog interactions: Assessing motivation and mental state in a randomized, controlled trial
Source: PLoS One. 2025 Jun 3;20(6):e0325325. doi: 10.1371/journal.pone.0325325 (PMC12133184; doi:10.1371/journal.pone.0325325)
Supplement: S1 Table — (ZIP) [file pone.0325325.s001.zip › Supporting information/Supporting information.docx]

Supporting information

Pairwise Fp1–Fp2

S1 Table. Pairwise comparisons for electrode pair Fp1–Fp2.

| **Contrast** | **Estimate** | **SE** | **df** | **t ratio** | **p value** |
| --- | --- | --- | --- | --- | --- |
| Baseline1–dog | 0.057 | 0.059 | 401 | 0.960 | 1 |
| Baseline1–neutral | 0.025 | 0.059 | 401 | 0.414 | 1 |
| Baseline1–plant | 0.062 | 0.059 | 401 | 1.046 | 1 |
| Baseline1–replica | 0.083 | 0.059 | 401 | 1.393 | 1 |
| Dog–neutral | −0.033 | 0.059 | 401 | −0.547 | 1 |
| Dog–plant | 0.005 | 0.059 | 401 | 0.086 | 1 |
| Dog–replica | 0.026 | 0.059 | 401 | 0.433 | 1 |
| Neutral–plant | 0.038 | 0.059 | 401 | 0.632 | 1 |
| Neutral–replica | 0.058 | 0.059 | 401 | 0.979 | 1 |
| Plant–replica | 0.021 | 0.059 | 401 | 0.347 | 1 |

Note. Statistics of the pairwise comparisons of the FAA scores between each condition for the electrode pair Fp1-Fp2. SE = standard error, df = degree of freedom.

Pairwise F3–F4

S2 Table: Pairwise comparisons for electrode pair F3–F4.

| **Contrast** | **Estimate** | **SE** | **df** | **t ratio** | **p value** |
| --- | --- | --- | --- | --- | --- |
| Baseline1–dog | −0.038 | 0.1 | 400 | −0.614 | 1 |
| Baseline1–neutral | 0.071 | 0.1 | 400 | 1.153 | 1 |
| Baseline1–plant | −0.060 | 0.1 | 400 | −0.966 | 1 |
| Baseline1–replica | −0.071 | 0.1 | 400 | −1.152 | 1 |
| Dog–neutral | 0.110 | 0.1 | 400 | 1.764 | 0.785 |
| Dog–plant | −0.022 | 0.1 | 400 | −0.349 | 1 |
| Dog–replica | −0.033 | 0.1 | 400 | −0.534 | 1 |
| Neutral–plant | −0.131 | 0.1 | 400 | −2.119 | 0.347 |
| Neutral–replica | −0.143 | 0.1 | 400 | −2.305 | 0.217 |
| Plant–replica | −0.012 | 0.1 | 400 | −0.186 | 1 |

Note. Statistics of the pairwise comparisons of the FAA scores between each condition for the electrode pair F3-F4. SE = standard error, df = degree of freedom.

Pairwise F7–F8

S3 Table: Pairwise comparisons for electrode pair F7–F8.

| **Contrast** | **Estimate** | **SE** | **df** | **t ratio** | **p value** |
| --- | --- | --- | --- | --- | --- |
| Baseline1–dog | −0.110 | 0.07 | 395 | −1.565 | 1 |
| Baseline1–neutral | 0.009 | 0.07 | 395 | 0.129 | 1 |
| Baseline1–plant | 0.060 | 0.07 | 395 | 0.853 | 1 |
| Baseline1–replica | −0.050 | 0.07 | 395 | −0.713 | 1 |
| Dog–neutral | 0.119 | 0.07 | 395 | 1.699 | 0.902 |
| Dog–plant | 0.171 | 0.07 | 395 | 2.414 | 0.162 |
| Dog–replica | 0.060 | 0.07 | 395 | 0.848 | 1 |
| Neutral–plant | 0.051 | 0.07 | 395 | 0.727 | 1 |
| Neutral–replica | −0.059 | 0.07 | 395 | −0.843 | 1 |
| Plant–replica | −0.111 | 0.07 | 395 | −1.562 | 1 |

Note: Statistics of the pairwise comparisons of the FAA scores between each condition for the electrode pair F7-F8. SE = standard error, df = degree of freedom.

S4 Table. Pairwise Comparisons and Effect Sizes for Interest Subscale

| Contrast | Estimate | SE | p_value | Effect_Size |
| --- | --- | --- | --- | --- |
| Dog - Plant | 2.588 | 0.145 | <0.001 | 2.704 |
| Dog - Replica | 2.405 | 0.145 | <0.001 | 2.513 |
| Plant - Replica | -0.182 | 0.145 | 0.63 | -0.191 |

Note. Estimates, standard errors, and p-values are presented for each comparison. The effect sizes are represented as Cohen’s d. Pairwise comparisons were adjusted using the Bonferroni correction method.

S5 Table. Pairwise Comparisons and Effect Sizes for Value Subscale

| Contrast | Estimate | SE | p_value | Effect_Size |
| --- | --- | --- | --- | --- |
| Dog - Plant | 2.099 | 0.146 | <0.001 | 2.175 |
| Dog - Replica | 2.217 | 0.146 | <0.001 | 2.298 |
| Plant - Replica | 0.118 | 0.146 | 1 | 0.123 |

Note. Estimates, standard errors, and p-values are presented for each comparison. The effect sizes are represented as Cohen’s d. Pairwise comparisons were adjusted using the Bonferroni correction method.

S6 Table. Pairwise Comparisons and Effect Sizes for Choice Subscale

| Contrast | Estimate | SE | p_value | Effect_Size |
| --- | --- | --- | --- | --- |
| Dog - Plant | 0.744 | 0.122 | <0.001 | 0.928 |
| Dog - Replica | 0.626 | 0.122 | <0.001 | 0.781 |
| Plant - Replica | -0.118 | 0.122 | 1 | -0.147 |

Note. Estimates, standard errors, and p-values are presented for each comparison. The effect sizes are represented as Cohen’s d. Pairwise comparisons were adjusted using the Bonferroni correction method.

S7 Table. Pairwise Comparisons and Effect Sizes for GB Subscale

| **Contrast** | **Estimate** | **SE** | **p_value** | **Effect_Size** |
| --- | --- | --- | --- | --- |
| Baseline - Dog | -0.655 | 0.174 | 0.001 | -0.572 |
| Baseline - Plant | 0.207 | 0.174 | 1.000 | 0.181 |
| Baseline - Replica | 0.103 | 0.174 | 1.000 | 0.090 |
| Dog - Plant | 0.862 | 0.174 | <0.001 | 0.752 |
| Dog - Replica | 0.759 | 0.174 | <0.001 | 0.662 |
| Plant - Replica | -0.103 | 0.174 | 1.000 | -0.090 |

Note. Estimates, standard errors, and p-values are presented for each comparison. The effect sizes are represented as Cohen’s d. Pairwise comparisons were adjusted using the Bonferroni correction method.

S8 Table. Pairwise Comparisons and Effect Sizes for WT Subscale

| **Contrast** | **Estimate** | **SE** | **p_value** | **Effect_Size** |
| --- | --- | --- | --- | --- |
| Baseline - Dog | -0.954 | 0.33 | 0.025 | 0.928 |
| Baseline - Plant | 1.506 | 0.33 | <0.001 | 0.781 |
| Baseline - Replica | 0.391 | 0.33 | 1.000 | -0.147 |
| Dog - Plant | 2.460 | 0.33 | <0.001 | 0.928 |
| Dog - Replica | 1.345 | 0.33 | <0.001 | 0.781 |
| Plant - Replica | -1.115 | 0.33 | 0.005 | -0.147 |

Note. Estimates, standard errors, and p-values are presented for each comparison. The effect sizes are represented as Cohen’s d. Pairwise comparisons were adjusted using the Bonferroni correction method.

S9 Table. Pairwise Comparisons and Effect Sizes for CR Subscale

| **Contrast** | **Estimate** | **SE** | **p_value** | **Effect_Size** |
| --- | --- | --- | --- | --- |
| Baseline - Dog | -1.034 | 0.225 | <0.001 | -0.696 |
| Baseline - Plant | -0.575 | 0.225 | 0.067 | -0.387 |
| Baseline - Replica | -0.310 | 0.225 | 1.000 | -0.209 |
| Dog - Plant | 0.460 | 0.225 | 0.253 | 0.309 |
| Dog - Replica | 0.724 | 0.225 | 0.009 | 0.487 |
| Plant - Replica | 0.264 | 0.225 | 1.000 | 0.178 |

Note. Estimates, standard errors, and p-values are presented for each comparison. The effect sizes are represented as Cohen’s d. Pairwise comparisons were adjusted using the Bonferroni correction method.

**S1 Fig.** **Distribution of frontal alpha asymmetry (FAA) scores for electrode pair Fp1-Fp2.** For each of the four experimental conditions: Baseline 1, Dog, Replica, and Plant. Each point represents an individual participant's FAA score at a given session. The plot illustrates intra-individual variability and potential condition-related trends across sessions. Participant 11 was excluded and is therefore not illustrated in the plot.

**S2 Fig.** **Distribution of frontal alpha asymmetry (FAA) scores for electrode pair F3-F4**. For each of the four experimental conditions: Baseline 1, Dog, Replica, and Plant. Each point represents an individual participant's FAA score at a given session. The plot illustrates intra-individual variability and potential condition-related trends across sessions. Participant 11 was excluded and is therefore not illustrated in the plot.

**S3 Fig.** **Distribution of frontal alpha asymmetry (FAA) scores for electrode pair F7-F8**. For each of the four experimental conditions: Baseline 1, Dog, Replica, and Plant. Each point represents an individual participant's FAA score at a given session. The plot illustrates intra-individual variability and potential condition-related trends across sessions. Participant 11 was excluded and is therefore not illustrated in the plot.

**S4 Fig. Correlation of FAA scores for electrode pair Fp1–Fp2 and IMI scores for each condition and subscale**. FAA = frontal alpha asymmetry IMI = intrinsic motivation inventory; r = Pearson r.

**S5 Fig. Correlation of FAA scores for electrode pair F3–F4 and IMI scores for each condition and subscale.** FAA = frontal alpha asymmetry; IMI = intrinsic motivation inventory; r = Pearson r.

**S6 Fig. Correlation of FAA scores for electrode pair F7–F8 and IMI scores for each condition and subscale.** FAA = frontal alpha asymmetry; IMI = intrinsic motivation inventory; r = Pearson r.

S10 Table: Correlations between interaction time of participants and dog in each session with FAA score for each electrode pair.

|  |  | Electrode pair | | |
| --- | --- | --- | --- | --- |
|  |  | Fp1–Fp2 | F3–F4 | F7–F8 |
| Session | 1 | 0.01 | 0.013 | 0.076 |
|  | 2 | 0.017 | −0.073 | −0.055 |
|  | 3 | −0.118 | 0.124 | −0.175 |

Note. Correlations represented as Pearson’s r.
