# Supplementary figures and images for: Neuromechanisms and subjective experiences during human-dog interactions: Assessing motivation and mental state in a randomized, controlled trial
Source: PLoS One. 2025 Jun 3;20(6):e0325325. doi: 10.1371/journal.pone.0325325 (PMC12133184; doi:10.1371/journal.pone.0325325)

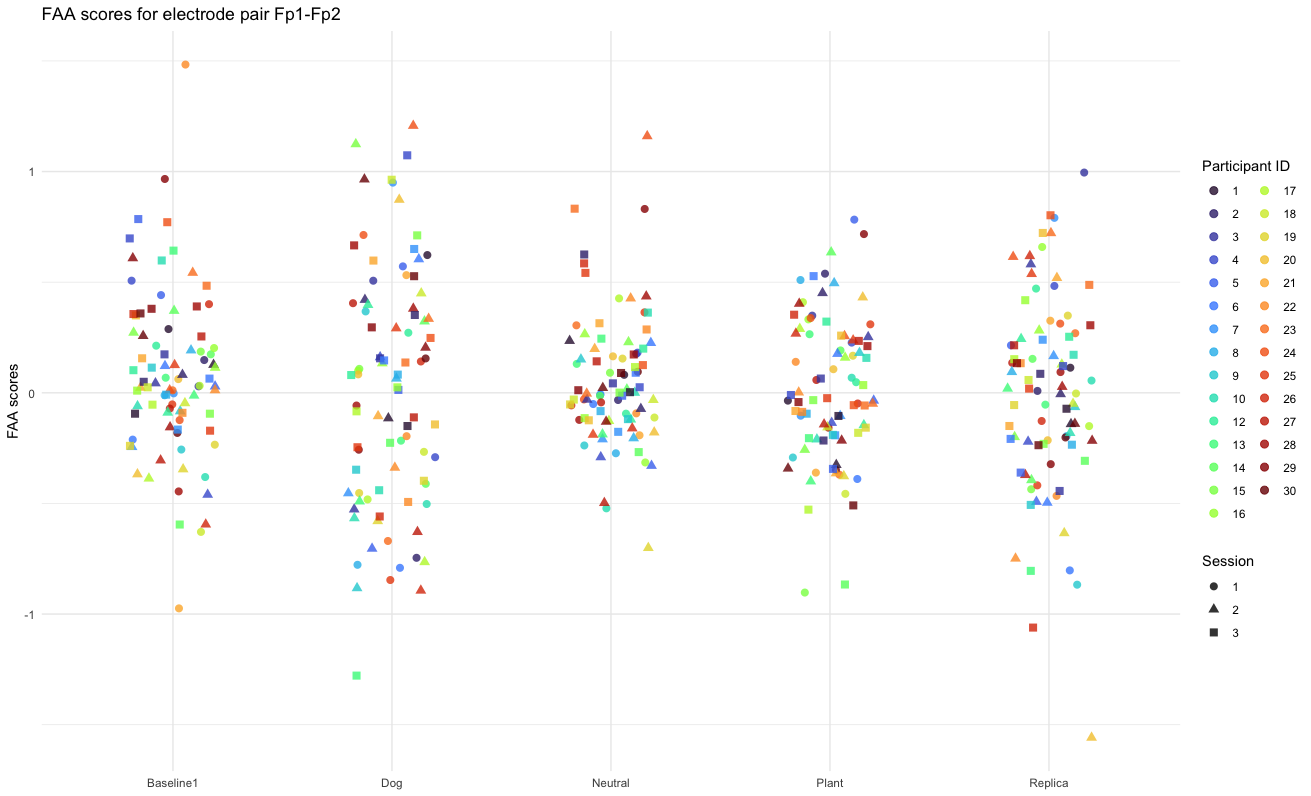

Supplement: S1 Table — (ZIP) [file pone.0325325.s001.zip › Supporting information/Fig S1.tif]

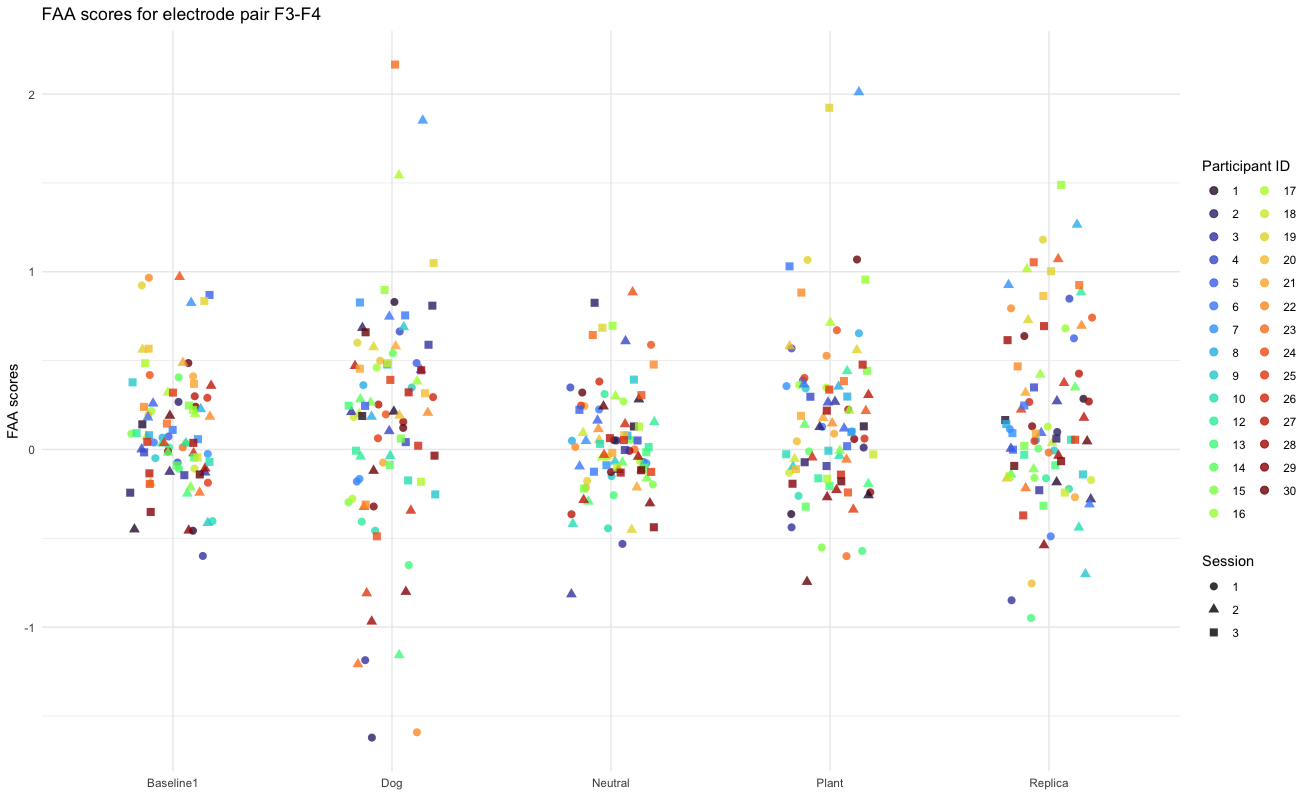

Supplement: S1 Table — (ZIP) [file pone.0325325.s001.zip › Supporting information/Fig S2.tif]

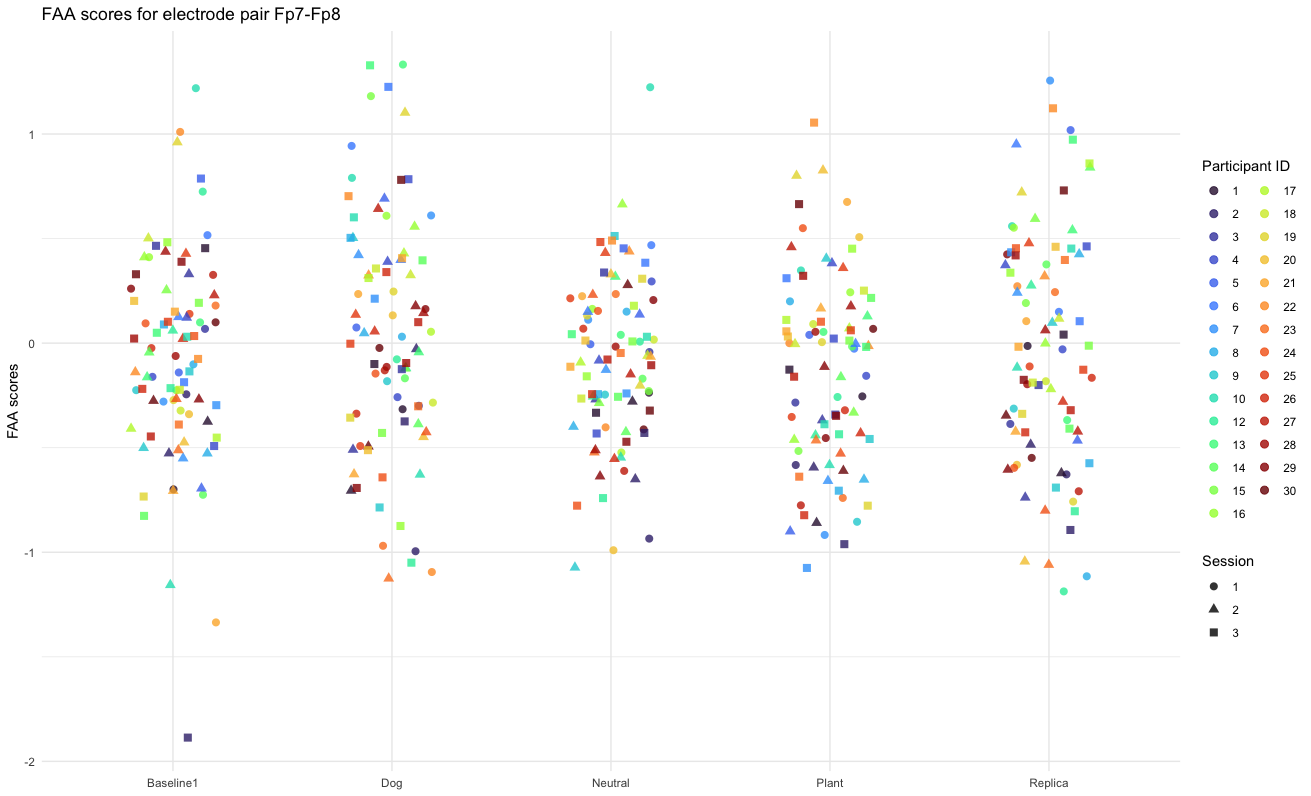

Supplement: S1 Table — (ZIP) [file pone.0325325.s001.zip › Supporting information/Fig S3.tif]

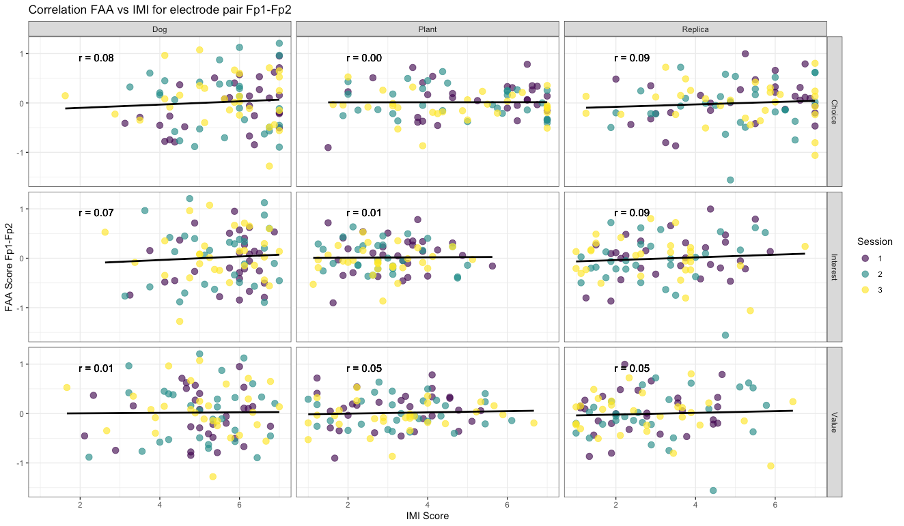

Supplement: S1 Table — (ZIP) [file pone.0325325.s001.zip › Supporting information/Fig S4.tif]

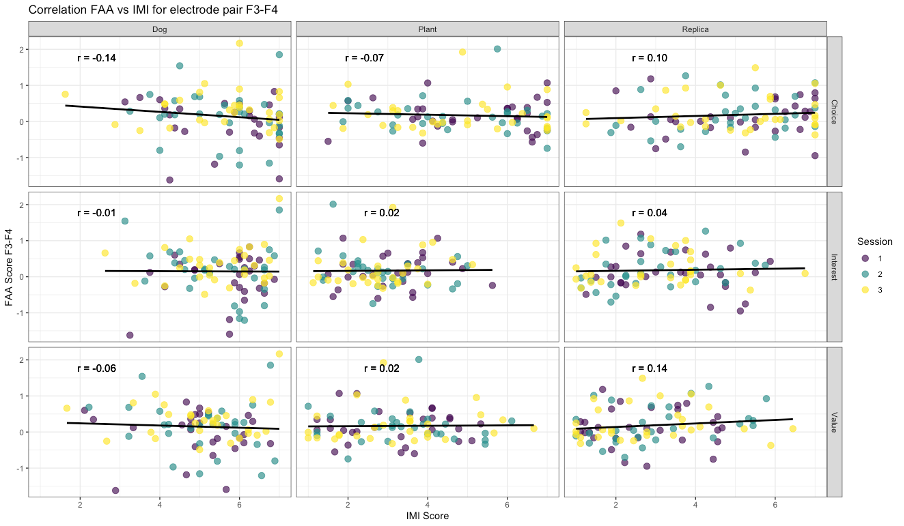

Supplement: S1 Table — (ZIP) [file pone.0325325.s001.zip › Supporting information/Fig S5.tif]

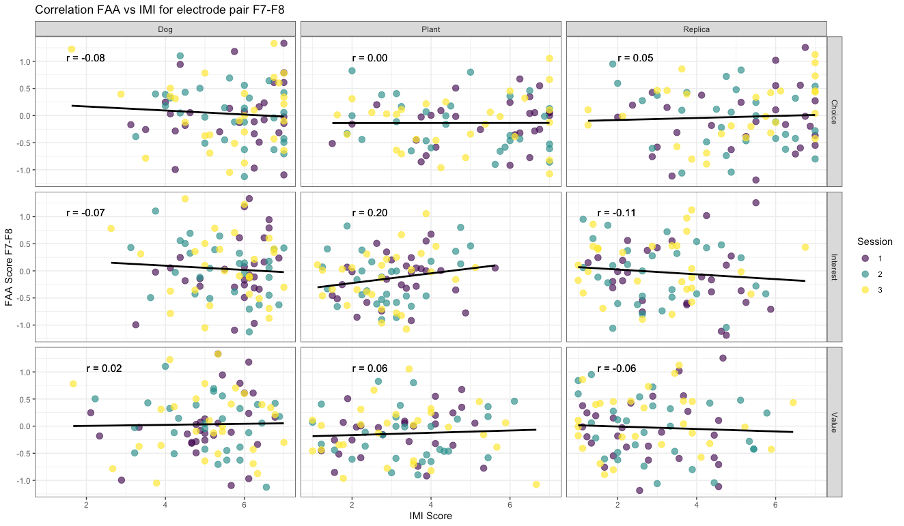

Supplement: S1 Table — (ZIP) [file pone.0325325.s001.zip › Supporting information/Fig S6.tif]
