# Supplementary material for: Neuromechanisms and subjective experiences during human-dog interactions: Assessing motivation and mental state in a randomized, controlled trial
Source: PLoS One. 2025 Jun 3;20(6):e0325325. doi: 10.1371/journal.pone.0325325 (PMC12133184; doi:10.1371/journal.pone.0325325)
Supplement: Attachment — Submitted filename: comments.docx [file pone.0325325.s017.docx]

**I have thoroughly seen this article. The idea is unique, but there are some comments which should be considered for screening the article in a good way.**

**Comments-1** The author must have to provide proper updated reference in many statement the consistent and contrast references are missing which making the study weaker.

**Comments-2** Proper procedure of randomization is not followed in the trial.

**Comments-3** For trial based study it is mandatory to specify the inclusion and exclusion criteria for the subjects under study in each groups.

**Comments-4** Although qualitative and quantitative touch is given in the study but it is not statistically significantly discussed in the abstract of the manuscript.

**Comments-5** If the author discussed about intervention in various groups but it not properly discussed in the manuscript and for intervention proper role of randomization is mandatory to avoid biasness. So the author has to scientifically clarify the matter. Thanks

**Comments-6** In line number 161 its coated that “Recruitment was conducted via online advertisements” it’s not clear that which online software and AI tool was used for the said procedure.

**Comments-7** Line number 233 -236 statement need proper consistent reference to support the current study please.

**Comments-8** The author must have to properly explain the type and structure of questionnaires which is implicated in the study.

**Comments-9** In line number 331 the author used IMI questionnaire please clear and explain what type and how many variety of questionnaires were used in the trial.

**Comments-10** Which specie of the plant you have used in this trial?

**Commetns-11** is there any relation of Dogs breeds in this experiment? In my point of view every breed have different characteristics

**Comments-12** Which type of stress you have focused in this trail?
